# Supplementary material for: Daily Profile of miRNAs in the Rat Colon and In Silico Analysis of Their Possible Relationship to Colorectal Cancer
Source: Biomedicines. 2025 Jul 31;13(8):1865. doi: 10.3390/biomedicines13081865 (PMC12383367; doi:10.3390/biomedicines13081865)
Supplement: Supplementary file 1 [file biomedicines-13-01865-s001.zip › biomedicines-3763672 Supplementary figures.pdf]

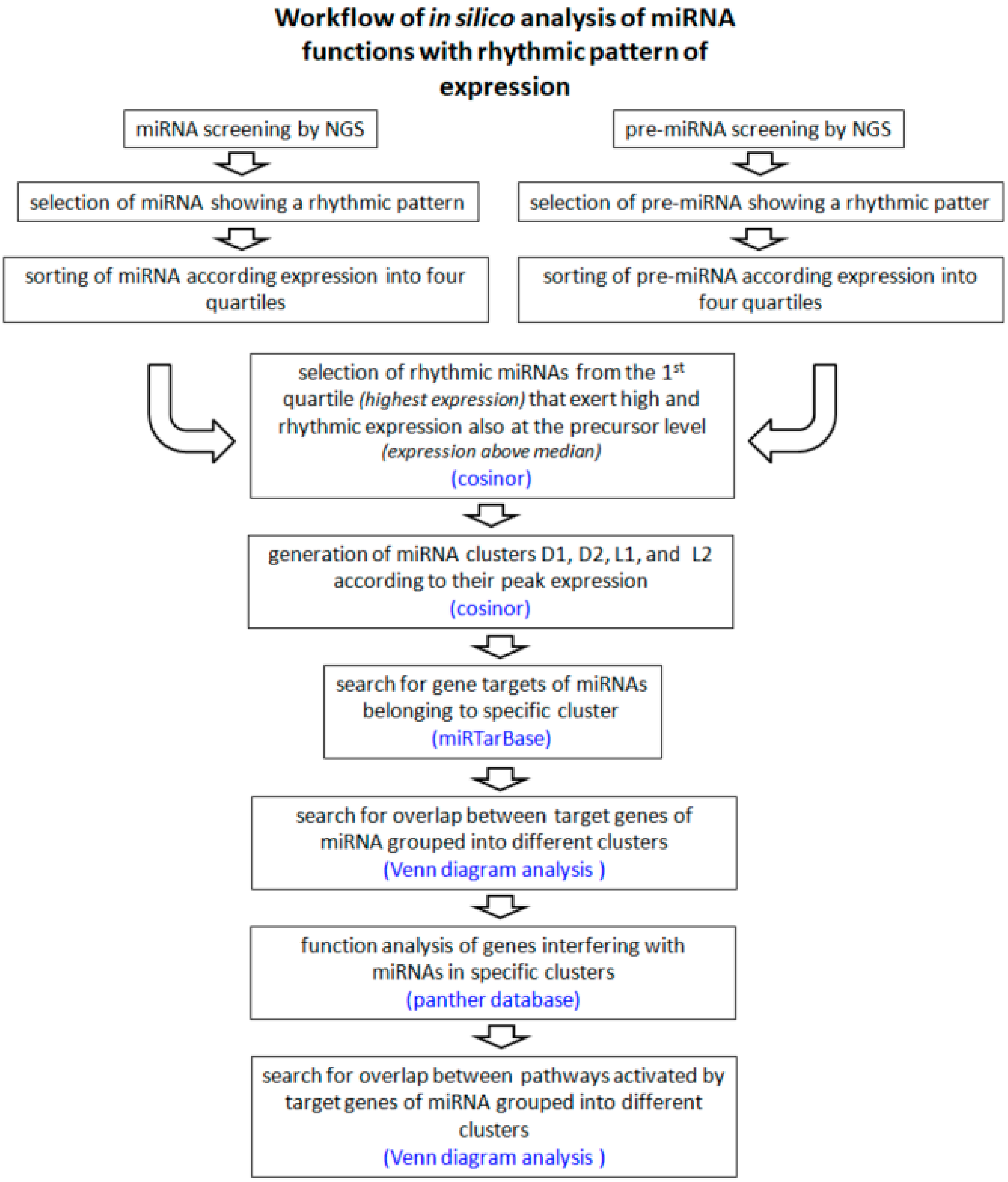

Figure S1

Workflow of *in silico* analysis of miRNA functions with rhythmic pattern of expression. NGS - Next Generation Sequencing; D1 – miRNAs with maximum expression during the first half of dark phase of LD cycle, D2 – miRNAs with maximum expression during the second half of dark phase of LD cycle; L1 – miRNAs with maximum expression during the first half of light phase of LD cycle, L2 – miRNAs with maximum expression during the second half of light phase of LD cycle.

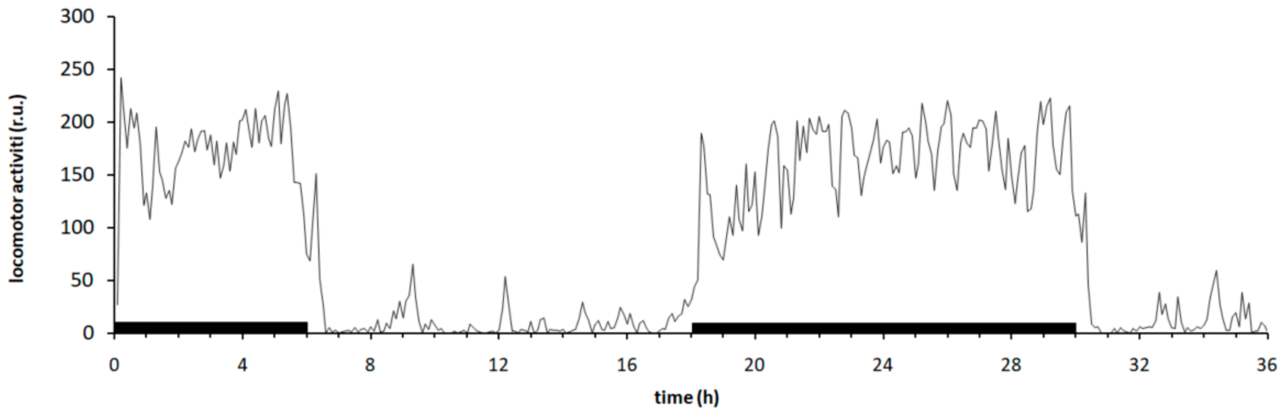

Figure S2

Locomotor activity of rats synchronized to light (L) :dark (D) cycle 12:12. Black bar at the bottom of the graph implicates dark phase of LD cycle; r.u. – relative units

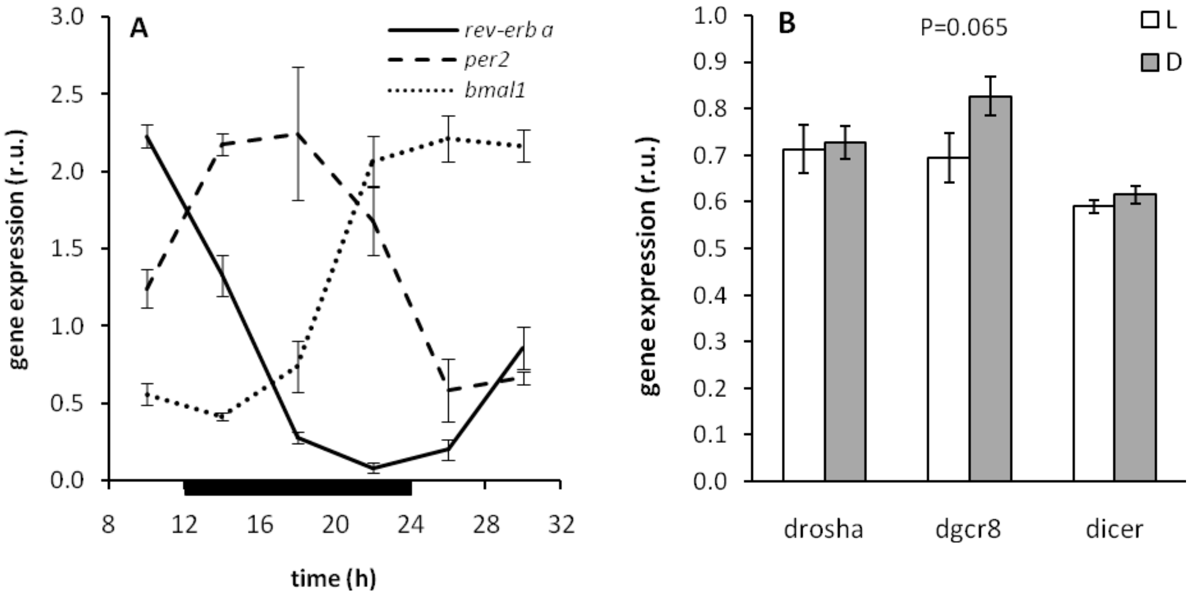

Figure S3

Daily pattern of *per2*, *bmal1* and *rev-erbα* expression in the rat colon (A; n = 4 - 5) and mRNA levels of *drosha*, *dgcr8* and *dicer* light (L) :dark (D) cycle 12:12 (B; n = 12 – 13). Data are presented as mean ± SEM. L – light phase, D – dark phase of LD cycle. r.u. – relative units
