# Supplementary material for: Daily Profile of miRNAs in the Rat Colon and In Silico Analysis of Their Possible Relationship to Colorectal Cancer
Source: Biomedicines. 2025 Jul 31;13(8):1865. doi: 10.3390/biomedicines13081865 (PMC12383367; doi:10.3390/biomedicines13081865)
Supplement: Supplementary file 1 [file biomedicines-13-01865-s001.zip › biomedicines-3763672 Table S1.pdf]

Table S1. Clinicopathological characteristics of the patient cohort

**All patients**	**47 (n)**	**100 (%)**
**Gender**		
Male	27	57.4
Female	20	42.6
**Age**<br>years (range, mean ± SEM)		
Younger (≤ median)<br>(37 - 68, 58.42 ± 8.46)	24	51.1
Older (>median)<br>(73 - 86, 79.35 ± 3.89)	23	48.9
**Tumour location**		
Right-side	19	40.4
Left-side	28	59.6
**Grading stage**		
G1	9	19.1
G2	35	74.5
G3	3	6.4
**Clinical stage**		
I	2	4.3
IIA, IIB	20	42.6
IIIA, IIIB	12	25.5
IVA, IVB	13	27.7
**TNM classification**<br>***Primary tumour invasion***		
T1-T2	2	4.3
T3	35	74.5
T4	10	21.3
Regional lymph node		
N0	25	53.2
N1	8	17.0
N2	14	29.8
Distant metastasis		
M0	34	72.3
M1	13	27.7

n = number, T = tumor invasion, N = nodal status, M = distant metastasis, SEM = standard error of the mean
