# Supplementary material for: Daily Profile of miRNAs in the Rat Colon and In Silico Analysis of Their Possible Relationship to Colorectal Cancer
Source: Biomedicines. 2025 Jul 31;13(8):1865. doi: 10.3390/biomedicines13081865 (PMC12383367; doi:10.3390/biomedicines13081865)
Supplement: Supplementary file 1 [file biomedicines-13-01865-s001.zip › biomedicines-3763672 Table S2.pdf]

Table S2 – Sequences of the primers used in real-time PCR

Gene	Strand	Sequence	Accession number
β-actin	Forward	5′-GGACTTCGAGCAAGAGATGG-3′	NM_001101.5
	Reverse	5′-GACTCCATGCCCAGGAAGG-3′	
bcl2	Forward	5′- TTCTTTGAGTTCGGTGGGGT -3′	NM_000633.3
	Reverse	5′- CTTCAGAGACAGCCAGGAGA -3′	
bmal1	Forward	5′-ACTTCCCCTCTACCTGCTCAA-3′	NM_001297724.1
	Reverse	5′-TGTCTTCATCCAGCCCCATC-3′	
cry1	Forward	5′- CCGTCTGTTTGTGATTCGTG -3′	NM_004075.4
	Reverse	5′- AAGTTAGAGGCGGTTGTCCA -3′	
cry2	Forward	5′- GGAGGCTGGTGTGGAAGTAG -3′	NM_001127457.2
	Reverse	5′- CGTAGGTCTCGTCGTGGTTC -3′	
dgcr8	Forward	5′-AAGAAGAGGCGAATGGAGGA-3′	NM_001105865.1
	Reverse	5′-CCCCAAGAAGTAGGGTCTGG-3′	
dicer	Forward	5′-ATGAGAAGCAAAAAGGTCAGCA-3′	XM_039113358.1
	Reverse	5′-ACATAACCAGGAGGAAGCCAA-3′	
drosha	Forward	5′-GCAGGAGACCCACAATACCAA-3′	NM_001107655.2
	Reverse	5′-AGAACGAATGCCAGTTTTCCA-3′	
myb	Forward	5′- AGTTCACTTGACCCACCCAA -3′	NM_001130173.2
	Reverse	5′- AGAACTGCGAGGGAGAGAAG -3′	
per2	Forward	5′-AATGCCGATATGTTTGCGGT-3′	NM_022817.1
	Reverse	5′-GCATCGCTGAAGGCATCTCT-3′	
rev-erb	Forward	5′-CAAGGCTGTCCCACCTACTTC-3′	NM_021724.5
	Reverse	5′-CCATTCAGCTTGGTGATGTTGC-3′	
rnu6-1	Forward	5′-GCTTCGGCAGCACATATACTAA-3′	NR_004394.1
	Reverse	5′-CGTTCAGCAAGTGAGCCAG-3′	
rnu6-2	Forward	5′- ATACAGAGAAGATTAGCATGGCC -3′	NR_125730.1

	Reverse	5′- CGAATTTGCGTGTCATCCTTG -3′	
snord47	Forward	5′- CCAATGATGTAATGATTCTGCCA -3′	NR_002746.1
	Reverse	5′- CCTCAGAATCAAAATGGAACGGT -3′	
miR-142-3p	Forward	5′-GACAGTGCAGTCACCCATA-3′	MIMAT0000434
	Reverse	5′-TAAAGTAGGAAACACTACACCCTCC-3′	
miR-30d-5p	Forward	5′-GTAAACATCCCCGACTGGAAGC-3′	MIMAT0000245
	Reverse	5′-GTAGCAGCAAACATCTGACTGAAAG-3′	
