# Supplementary material for: Daily Profile of miRNAs in the Rat Colon and In Silico Analysis of Their Possible Relationship to Colorectal Cancer
Source: Biomedicines. 2025 Jul 31;13(8):1865. doi: 10.3390/biomedicines13081865 (PMC12383367; doi:10.3390/biomedicines13081865)
Supplement: Supplementary file 1 [file biomedicines-13-01865-s001.zip › biomedicines-3763672 Table S4.pdf]

Table S4 – miRNAs sorted according to their peak expression

Acrophase	miRNA	Sequence	Species	Homology of	
				whole sequence	seed sequence
D1	rno-miR-128-3p	UCACAGUGAACCGGUCUCUUU	rat	100%	100%
	hsa-miR-128-3p	UCACAGUGAACCGGUCUCUUU	human		
D1	rno-miR-129-5p	CUUUUUGCGGUCUGGGCUUGC	rat	100%	100%
	hsa-miR-129-5p	CUUUUUGCGGUCUGGGCUUGC	human		
D1	rno-miR-139-5p	UCUACAGUGCACGUGUCUCCAG	rat	95.65%	100%
	hsa-miR-139-5p	UCUACAGUGCACGUGUCUCCAG~~U~~	human		
D1	rno-miR-150-5p	UCUCCCAACCCUUGUACCAGUG	rat	100%	100%
	hsa-miR-150-5p	UCUCCCAACCCUUGUACCAGUG	human		
D1	rno-miR-425-5p	AAUGACACGAUCACUCCCGUUGA	rat	100%	100%
	hsa-miR-425-5p	AAUGACACGAUCACUCCCGUUGA	human		
D2	rno-let-7g-5p	UGAGGUAGUAGUUUGUACAGUU	rat	100%	100%
	hsa let 7g 5p	UGAGGUAGUAGUUUGUACAGUU	human		
D2	rno-miR-148a-3p	UCAGUGCACUACAGAACUUUG	rat	95.45%	100%
	hsa-miR-148a-3p	UCAGUGCACUACAGAACUUUG~~U~~	human		
D2	rno-miR-148b-3p	UCAGUGCAUCACAGAACUUUGU	rat	100%	100%
	hsa-miR-148b-3p	UCAGUGCAUCACAGAACUUUGU	human		
D2	rno-miR-185-5p	UGGAGAGAAAGGCAGUUCCUGA	rat	100%	100%
	hsa-miR-185-5p	UGGAGAGAAAGGCAGUUCCUGA	human		
L1	rno-miR-30d-5p	UGUAAACAUCCCCGACUGGAAG	rat	100%	100%
	hsa-miR-30d-5p	UGUAAACAUCCCCGACUGGAAG	human		

D1 – maximum expression during the first half of dark phase of LD cycle, D2 – maximum expression during the second half of dark phase of LD cycle; L1 – maximum expression during the first half of light phase of LD cycle; underlined part of sequence shows seed sequence; strikethrough letters shows part of mature mRNA sequence which is not in human and rat identical.
