# Supplementary material for: Daily Profile of miRNAs in the Rat Colon and In Silico Analysis of Their Possible Relationship to Colorectal Cancer
Source: Biomedicines. 2025 Jul 31;13(8):1865. doi: 10.3390/biomedicines13081865 (PMC12383367; doi:10.3390/biomedicines13081865)
Supplement: Supplementary file 1 [file biomedicines-13-01865-s001.zip › biomedicines-3763672 Table S5.pdf]

Table S5 – Genomic context of rhythmic miRNAs clustered according to their acrophases

Acrophase	miRNA	Genomic context	Host gene	Localisation	Seed sequence
D1	hsa-miR-128-3p	intragenic	ARPP21, R3HDM	intron	broadly conserved
D1	hsa-miR-129-5p	intragenic	CALUMENIN 1	intron	broadly conserved
D1	hsa-miR-139-5p	intragenic	Gm45837	intron	broadly conserved
D1	hsa-miR-150-5p	intergenic			broadly conserved
D1	hsa-miR-425-5p	mixed			broadly conserved
D2	hsa-let-7g-5p	intragenic	WDR82	intron	broadly conserved
D2	hsa-miR-148b-3p	intragenic	COPZ1	intron	broadly conserved
D2	hsa-miR-148a-3p	intergenic			broadly conserved
D2	hsa-miR-185-5p	intragenic	TANGO2	intron	conserved
L1	hsa-miR-30d-5p	intergenic			broadly conserved

D1 – maximum expression during the first half of dark phase of LD cycle, D2 – maximum expression during the second half of dark phase of LD cycle; L1 – maximum expression during the first half of light phase of LD cycle; (Rodriguez et al., 2004; Bhattacharyya et al., 2012; Agarwal et al., 2015)
